# Social inequalities in childhood asthma^☆^

**DOI:** 10.1016/j.waojou.2024.101010

**Published:** 2024-12-03

**Authors:** Angela Pinot de Moira, Adnan Custovic

**Affiliations:** National Heart and Lung Institute, Imperial College London, London, United Kingdom

**Keywords:** Childhood asthma, Socioeconomic, Inequality/inequalities

## Abstract

Asthma is a complex, heterogeneous condition, broadly characterized by chronic airway inflammation with variable expiratory airflow limitation, but with several subtypes underpinned by different (although likely overlapping) pathological mechanisms. It is one of the most common chronic diseases of childhood and represents a significant cost for healthcare systems and affected families. Evidence suggests that a disproportionate proportion of this burden falls on families from disadvantaged socioeconomic circumstances (SECs).

In this review, we describe the extent to which growing up in disadvantaged SECs is associated with an increased risk of childhood asthma diagnosis and asthma outcomes, including how this differs geographically and across different asthma subtypes. We also discuss the complex and interdependent mediating pathways that may link disadvantaged SECs with childhood asthma and asthma-related outcomes.

In high-income countries (HICs), there is a fairly consistent association between growing up in disadvantaged SECs and increased prevalence of childhood asthma. However, evidence suggests that this social patterning differs across different asthma subtypes, with asthma phenotypes associated with disadvantaged SECs being less likely to be associated with atopy and more likely to begin in infancy and persist into adolescence. Disadvantaged SECs are also associated with worse asthma outcomes, which may contribute to the persistence of symptoms among disadvantaged children.

In low- and middle-income countries (LMICs), the patterns are more variable and data more limited, but there is some evidence that disadvantaged SECs and atopic asthma are similarly negatively associated. There are also clear disparities in asthma outcomes, with LMICs having disproportionately high asthma-related morbidity and mortality, despite having lower asthma prevalence. A lack of accessibility to essential medication and appropriate care no doubt contributes to these disparities.

The pathways leading to social inequalities in asthma are complex and interdependent, and as yet not fully understood. There is a clear need for further research into the relative importance of potential mediating pathways, including how these vary across the life course and across asthma subtypes. A stronger understanding of these pathways will help identify the most effective policy entry points for intervention, ultimately reducing inequalities across the life course.

## Introduction

Asthma is one of the most common chronic diseases of childhood, with global prevalence estimates of 9% for children and 11% for adolescents.^1^ It is characterized by chronic airway inflammation and symptoms such as wheeze, shortness of breath and cough, with variable expiratory airflow limitation.^2^ Importantly, it is recognized to be a complex, heterogeneous condition, with several subtypes underpinned by different pathological mechanisms, which likely overlap in some patients.^3^ Although these mechanisms are not yet fully understood, environmental and social conditions, particularly in early-life, play an important role in the development of different asthma subtypes.^4^

The condition is associated with substantial direct costs to health care systems,^5^^,^^6^ with 50% of these costs estimated to be spent on the ∼20% of individuals with severe symptoms and frequent exacerbations (attacks).^7^ Additionally, asthma incurs considerable indirect costs related to missed work and school days and premature mortality.^5^^,^^6^ Childhood asthma, in particular, contributes to greater indirect costs due to both the lost school days of the child and lost work days of the caregiver.^7^ Asthma also considerably impacts the quality of life of both the patient and their family.^8^ Furthermore, the impact of childhood asthma, particularly if symptoms are frequent or severe, extends beyond childhood, increasing the risk of impaired lung function, asthma and chronic obstructive pulmonary disease (COPD) in adulthood.^9^^,^^10^

Evidence shows that this burden of childhood asthma disproportionally falls on underserved families. Not only are children growing up in socioeconomically disadvantaged circumstances more likely to develop persistent asthma,^11^ they are also more likely to experience worse asthma outcomes^12^ and lower quality of asthma care.^13^ There is evidence, however, that the social patterning of asthma varies across countries and for different types of asthma.

In this review, we describe the extent of socioeconomic inequalities in asthma, including how these differ geographically and for different subtypes of asthma. We also discuss the pathways that may lead to inequalities in the development of asthma and asthma outcomes, and how these may cluster and interact to further exacerbate risks.

We focus on socioeconomic inequalities in asthma, as opposed to inequalities across different races/ethnicities/cultures/languages, genders/sexes, or religion, although recognise that these factors may contribute to socioeconomic inequalities. We use the term inequality, which refers to differences in the distribution of a specific factor (in this case childhood asthma diagnosis) between population groups, but also acknowledge that differences in the distribution of the disease across socioeconomic groups are largely avoidable, as well as unfair and unjust, and therefore could also be termed inequities in childhood asthma.^14^

## Social patterning of asthma prevalence

The evidence for an association between disadvantaged socioeconomic circumstances (SECs) and increased prevalence of asthma in high-income countries is fairly consistent. For instance in a systematic review of studies from mainly middle- and high-income countries, 63% of studies reported an association between lower SECs and asthma, with a pooled estimate of 36% increased risk of asthma among people with lower SECs.^15^ Meta-analyses of birth cohort data also suggest a consistent tendency for children from less advantaged SECs to be at greater risk of asthma. For example, in an analysis of 7 birth cohorts across 6 European countries, all cohorts showed a tendency towards greater risk of childhood asthma among children born to mothers with a lower versus higher education level, with risk ratios ranging between 1.07 (95%CI: 0.97–1.18) and 1.61 (95%CI: 1.08–2.40) (*I*^*2*^ = 21.6%).^16^ In another study conducted across 10 European countries, all cohorts except the Italian GASPII birth cohort displayed an association of increased relative and absolute risk among children born to mothers in the lowest compared with highest tertile of education (Relative and Slope Indices of Inequality ranged between 1.17-2.07 and 0.43–9.03, respectively).^17^ In this study, although there was low heterogeneity in relative inequalities (*I*^*2*^ = 9.2%), there was greater heterogeneity in absolute inequalities (*I*^*2*^ = 72.0%). Finally, a meta-analysis of 7 birth cohort studies across 3 continents (Europe—United Kingdom, the Netherlands and Sweden, North America—United States and Canada, and Oceania—Australia) also observed evidence for social inequalities in asthma-related outcomes, but with stronger associations for household income than for maternal education.^18^

Although there have also been studies from low- and middle-income countries (LMICs) describing inverse associations between indicators of higher socioeconomic conditions and asthma, particularly in Latin America,^19^, ^20^, ^21^, ^22^ these findings have not been consistent across LMIC studies, with positive associations having also been described.^23^ There also appears a rural-urban divide in LMICs,^24^^,^^25^ with higher prevalence of asthma in urban communities,^26^, ^27^, ^28^ but with evidence of increasing prevalence of asthma in rural populations.^25^ An ecological study in Ecuador observed a higher prevalence of asthma in communities transitioning from traditional rural to urban living, with indicators of higher socioeconomic conditions and urbanised lifestyle associated with greater asthma prevalence.^29^

## Social patterning of asthma subtypes

While individuals with an asthma diagnosis may present with similar symptoms such as wheeze, cough, shortness of breath, it is now recognized that these symptoms can arise from a variety of distinct subtypes, each with different pathophysiological mechanisms.^3^^,^^30^ There is evidence that subtypes of asthma can also differ in their social patterning. For example, evidence from birth cohort studies suggests that asthma without atopic symptoms is more strongly associated with early life disadvantaged SECs than asthma with atopy,^31^^,^^32^ which aligns with findings from systematic reviews indicating that atopy is associated with more advantaged SECs.^15^ Additionally, there is evidence that asthma associated with urbanization in LMICs is more likely to be linked with atopy,^33^ an association that has also been observed between rich and poor urban communities.^24^^,^^25^ For instance, a study conducted in a large city in Ghana observed an association between higher IgE titres to mite and asthma/wheezing among children attending an urban affluent school, but no association among children attending schools in urban poor and suburban/rural communities;^34^ similar results have been described elsewhere.^35^ A factor that has been proposed to contribute towards the lower prevalence of allergy in rural LMIC communities is their higher prevalence and intensity of helminth infections, which have been shown to down-regulate allergic responses and potentially decouple IgE from its effector mechanisms;^36^^,^^37^ however, other factors such as diet, type of drinking water,^38^ and environment are also likely to contribute.^39^

Provided that the mechanisms leading to allergic sensitization are also on the causal pathway to asthma,^40^ these differences in association with atopic-versus non-atopic asthma would suggest that the contribution of risk factors to the development of asthma may differ across social groups. Hence, to understand what factors are important in driving observed inequalities, it is important to consider asthma's heterogeneity.

An approach taken in research to discern potential subtypes of asthma is to examine longitudinal symptoms of asthma using birth cohort data. In a seminal study, Martinez et al used a hypothesis-driven approach to identify 3 subtypes of childhood asthma (early transient, late-onset, and persistent) based on temporal patterns of wheezing in the first 6 years of life.^41^ This approach has since been extended using data-driven, statistical machine learning techniques to uncover wheezing patterns, with typically 1^42^, ^43^, ^44^, ^45^ or 2^46^, ^47^, ^48^ further subtypes identified that represent later onset and/or intermittent patterns of wheeze. However, while this approach has the advantage of reducing the risk of investigator bias, it can result in subtypes that are not stable or internally homogenous, depending on the method applied.^49^ In addition, although subtypes are often assigned similar labels across studies, their characteristics may differ depending on study design, timing and frequency of data collection and sample size. Finally, results of similar studies in LMICs suggest different structures in data sets, and different associates of lung function^50^ and subtypes of wheezing^51^ compared to data from high-income countries.

With these caveats in mind, among the relatively few studies that have specifically examined how SECs associate with temporal patterns of symptoms, early-onset persistent wheeze has most consistently been associated with disadvantaged SECs, using both data-driven^32^^,^^52^ and hypothesis-driven approaches.^11^^,^^53^ Associations with disadvantaged SECs have also been observed for early transient^52^^,^^53^ and intermediate-onset subtypes,^52^ but less consistently across studies. There have been very limited studies from LMICs examining temporal patterns of asthma symptoms. However, 1 study conducted in the South African Drakenstein Child Health Study using partition around medoids clustering of 6 multi-dimensional variables of wheezing spells from birth to adolescence, observed a positive association between indicators of higher SECs and late onset wheeze in unadjusted analysis,^51^ suggesting potentially different social patterning compared to HICs.

Since preschool wheeze is often triggered by viral infection,^54^ and is also not specific to asthma but may be present with other paediatric conditions such as bronchiolitis,^55^ respiratory tract infections, and congenital airway anomalies (themselves socially patterned), it is unclear to what extent the consistent association of disadvantaged SECs with early-onset persistent symptoms of asthma in HICs is reflective of a single persistent subtype which has its origins in early life, vs separate, dynamic subtypes, displaying developmental changes over time. There is evidence that respiratory tract infections play a role in driving inequalities in chronic asthma, but it is not known how this differs across asthma subtypes.^52^ In addition, since the above approaches for subtyping asthma focus on the presence or absence of wheeze, it is also possible that the increased risk of persistent symptoms among children from disadvantaged SECs is partly driven by exacerbating environmental factors and/or inadequate access to healthcare. This was observed in a New Zealand study conducted among Māori and non-Māori children where incidence of asthma was similar in the 2 ethnic groups, but Māori children were more likely to experience persistent symptoms into adolescence and adulthood, partly due to environmental factors, but also due to inadequate access to appropriate health care and asthma education.^56^

## Social patterning of asthma outcomes

Other studies have similarly demonstrated barriers to healthcare and worse asthma outcomes among children from disadvantaged SECs. For example, in a Danish registry study, children from the lowest income quartile were less likely to redeem a prescription for asthma medication but were more likely to have an asthma-related hospitalization.^57^ Similarly, another Danish study observed an association between higher parental education and income with reduced risk of uncontrolled asthma and exacerbations.^58^ Exacerbations pose the greatest risk to children with asthma, can occur regardless of severity or frequency of symptoms,^59^, ^60^, ^61^ and are associated with persistently lower lung function^62^^,^^63^ and greater risk of COPD in midlife.^10^ A systematic review examining risk factors for exacerbations in children aged 5–12 years reported a positive association between poverty and the risk of attacks in 7 out of 9 identified studies, with odds ratios between 1.4 and 2.8. Additionally, an increased risk of exacerbations was observed among children with low parental education compared to those with high parental education in all 4 identified studies, with odds ratios ranging from 1.1 to 1.9.^64^

Several studies have also described an increased risk of asthma-related emergency department (ED) visits,^65^ hospital admissions,^65^, ^66^, ^67^ serious asthma,^68^ and intensive care admissions^12^^,^^67^ and readmissions^69^ associated with disadvantaged SECs. A systematic review of studies examining socioeconomic disparities (relating to income, poverty, education, employment status, or health care insurance coverage) in asthma health care utilization and outcomes, reported a reduced risk of primary care attendance (OR = 0.52 (95% CI: 0.45, 0.60), *I*^2^ < 0.01), but a consistently increased risk of ED visits (OR = 1.54 (95% CI: 1.25, 1.90), *I*^2^ = 53.4), hospital admission (OR = 1.66 (95% CI: 1.24, 2.23), *I*^2^ = 62.0) and readmission (OR = 1.37 (1.16, 1.63), *I*^2^ = 27.8) associated with disadvantaged SEC in paediatric studies.^70^

Within LMICs, asthma management is severely limited by a lack of availability of essential medication. Data are limited, but a systematic review of studies published between 2010 and 2022 found that only 6 out of 23 LMICs reporting availability of short-acting beta-agonists (SABA) reached the World Health Organisation (WHO) target goal of achieving 80% availability in public and private facilities; only 1 out of 22 LMICs reporting availability of inhaled corticosteroids (ICS) reached the WHO target.^71^ The review also found that the majority of studies reported medication costs of 1–4 days' salary for 1 month of SABA, and 2–7 days’ salary for 1 month of ICS. In addition to restricted availability and affordability of essential asthma medication, there is also limited availability of spacer devices and suboptimal management of acute exacerbations and diagnosis of asthma in LMICs (reviewed in Mortimer et al^72^). Unfortunately, there is also a lack of research into the effectiveness and feasibility of implementing new asthma treatments in LMIC populations, which is likely to result in a widening care gap.^72^ These inequalities are reflected in the global patterns of disease: while there is a clear trend of increasing prevalence of asthma with increasing country wealth,^1^^,^^73^ prevalence of severe symptoms and asthma morality rates follow the opposite trend, with greater morbidity and mortality in LMICs.^72^^,^^74^

## Drivers of inequalities

In order to establish potential interventions aimed at addressing social inequalities in childhood asthma, it is important to understand the mediating pathways that link disadvantaged SECs with childhood asthma and asthma-related outcomes. Various pathways to health inequalities have been proposed, including those related to material resources, psychosocial elements, health behaviors, and structural components that influence the distribution and access to power, resources, and services among different populations (reviewed in Pearce et al^75^), all of which are likely to contribute to social inequalities in childhood asthma.

It is also important to consider how these mediating pathways operate across the life course. For example, the pre- and post-natal periods will be critical and sensitive periods for the effects of exposures associated with disadvantaged SECs due to the rapid development of the respiratory and immune systems during this time. A critical period refers to a specific window during an individual's development when an exposure can significantly affect development and subsequent health; outside this window the same exposure may not have the same effect.^76^ For instance, nicotine exposure *in utero* increases the risk of low birth weight and pre-term birth,^77^^,^^78^ altered lung^79^ and immune^80^^,^^81^ development, and exacerbates the risks associated with subsequent exposures, all impacting later lung health.

A sensitive period refers to a window when an exposure may have a stronger effect on development and health than at other times, but may still have an effect outside this window.^76^ For example, early-life may represent a sensitive period for allergic sensitization or immune tolerance, with multiple early sensitization strongly increasing the risk of developing asthma.^82^, ^83^, ^84^, ^85^ Similarly, diversification of the human microbiome around the time of birth is thought essential for the normal development of the immune system,^86^, ^87^, ^88^, ^89^ including transitioning away from a tolerogenic, T helper 2-skewed immune response, towards a protective immune response,^90^, ^91^, ^92^ which may be accelerated by maternal exposures during pregnancy.^93^, ^94^, ^95^ Conversely, exposure to antibiotics during pregnancy and early life, which has been observed to be greater among socioeconomically disadvantaged mothers and children,^96^, ^97^, ^98^, ^99^ may alter the development of the microbiome.^100^ Early life antibiotic exposure has also been associated with an increased risk of asthma,^101^ although this relationship may be partly confounded by an increased propensity for infections.^102^

Accumulated risks over the life course occurring independently of any developmental stage are also likely to contribute to social inequalities in asthma, reflected by observations that persistent poverty, as opposed to transient poverty, associates more strongly with higher asthma prevalence.^103^^,^^104^ Children growing up in socioeconomically disadvantaged circumstances are at increased risk of cumulative detrimental exposures over their life course, from maternal smoking during pregnancy,^105^ low birth weight and preterm birth,^16^^,^^105^, ^106^, ^107^ to poorer housing conditions^108^ and poorer diets,^109^ all of which will increase their risk of developing asthma and worse asthma outcomes.^110^, ^111^, ^112^

Exposures at 1 stage of the life course may also increase susceptibility or sensitivity to exposures at other stages, resulting in a so-called “chain of risk”.^76^ For example, poor families are at increased risk of fuel poverty, whereby a family cannot afford to heat their home to an adequate temperature. As well as increasing respiratory symptoms among individuals with asthma,^113^ cold homes also increase susceptibility to respiratory tract infections by increasing the transmissibility and viability of viruses,^114^ and potentially also affecting host susceptibility and innate immune responses to respiratory viral infections.^115^ Respiratory tract infections in turn increase the risk of developing asthma and impaired lung function in childhood,^116^ as well as asthma exacerbations in children with asthma.^117^ Additionally, since cold air is able to hold less moisture,^118^ condensation is more likely to form in colder homes, creating an environment that encourages mould and damp, which are similarly associated with an increased risk of the development and exacerbations of asthma.^119^ Furthermore, poor housing conditions and cold homes negatively impact mental health, which may increase inflammatory reactivity to environmental stressors such as indoor and outdoor air pollution.

## Identifying targets for intervention

**Fig. 1:**
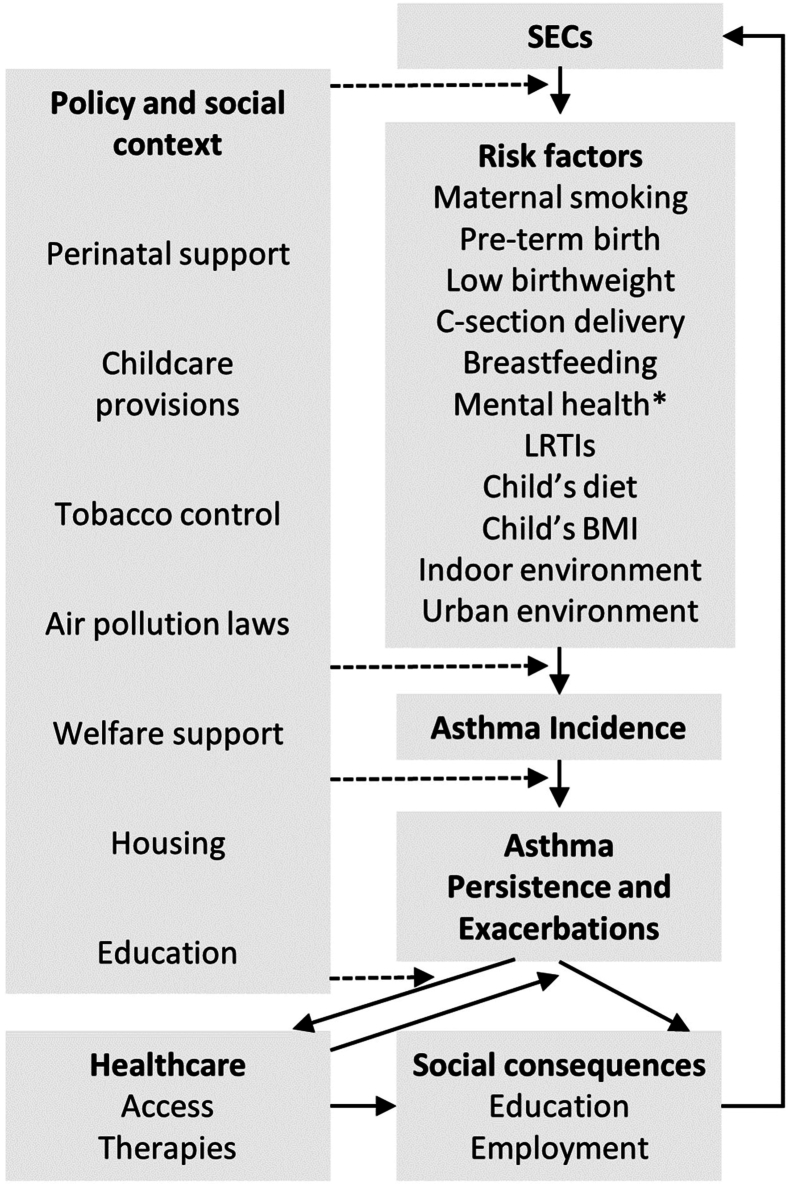
**Adapted Diderichsen model of pathways to socioeconomic inequalities in childhood asthma**. ∗Maternal and child mental health. Abbreviations: *SECs* – socioeconomic circumstances; *LRTIs* – lower respiratory tract infections.

The above highlights the complexity and inter-dependence of the mediating pathways that link disadvantaged SECs with childhood asthma and asthma-related outcomes. Diderichsen et al. provide a framework for understanding these intricate pathways and identifying possible policy entry points for intervention (Fig. 1).^120^ This begins with social stratification itself - the hierarchical arrangement of individuals in a society based on factors like socioeconomic status, power, and access to resources, which collectively influence health and the distribution of health outcomes within a population. Policy interventions aimed at this stage will focus on creating a more equitable society through measures such as universal access to healthcare, progressive taxation, and ensuring equal opportunities for education and employment. The effects of social position on health are then mediated by the uneven distribution (differential exposure) and different effects (differential susceptibility) of the causes of disease (in this case childhood asthma) across social groups. Health inequalities may in turn be perpetuated by differences in the social and financial repercussions of ill-health across social groups (differential consequences). For instance, childhood asthma has been linked with higher rates of school absenteeism, particularly among children from disadvantaged SECs^121^^,^^122^ and can impact educational attainment,^123^^,^^124^ thus contributing to persistent inequalities across generations.

### Targets for intervention: prevention of asthma development

Modern counterfactual mediation methods provide a useful tool for obtaining unbiased estimates of differential exposure (total indirect effect^125^) and differential susceptibility (reference interaction and mediated interaction^125^).^126^ However, to date, relatively few studies have applied these methods to investigate the mediating pathways to social inequalities in childhood asthma. Lewis et al used Scottish registry data to examine associations of Scottish Index for Multiple Deprivation and the mediating role of bronchiolitis in inequalities in chronic asthma; their findings suggested bronchiolitis mediated 18%–21% of observed risk differences across deprivation groups.^52^ In the UK Millennium Cohort Study, Creese et al examined how blocks of factors mediate the total effect of maternal education on persistent wheeze, with findings suggesting that perinatal factors (including maternal pre-pregnancy BMI, breastfeeding duration, birth weight, smoking and alcohol use during pregnancy, and childhood wheeze) mediate 37.5% of observed inequalities, and both perinatal and home environment factors 61.2% of inequalities.^11^ A more recent study examined how perinatal factors (maternal smoking, birthweight, gestational age, caesarean delivery and breastfeeding) mediate the relationship between early-life SECs and childhood asthma in 7 birth cohorts across 6 European countries. The study found a relatively consistent mediated effect across cohorts (relative risk range: 1.03–1.09), but observed variation in the proportion mediated (8–72%).^16^ This suggests that while improved perinatal health may have a similar effect on reducing the risk of asthma among children from disadvantaged SECs across countries, the relative reduction in inequalities will vary. Additionally, the study found some evidence for differential susceptibility to mediators across social groups in Scandinavian countries. There remains, however, a number of potential mediators that have not been examined, such as ambient air pollution, access to green and blue space, diet and maternal and child mental health.

### Targets for intervention: improving asthma outcomes

Very few, studies have applied these methods to examine the mediators of inequalities in asthma outcomes such as exacerbations and hospital admissions among children with asthma. Those that have, have mostly focused on race rather than SECs. Using a structural equation modelling framework (as opposed to counterfactual mediation methods), Correa-Agudelo et al examined to what extent individual- and neighbourhood-level variables explain observed associations between race (as opposed to SECs) and asthma-related emergency department visits at Cincinnati Children's Hospital Medical Center, Ohio. They found that medical insurance, neighbourhood socioeconomic deprivation index and exposure to particulate matter with a diameter less than 2.5 μm (PM_2.5_) and outdoor mould accounted for 55% of the effect of race on number of ED visits.^127^ In a separate analysis, family hardship was observed to explain 40% of the effect of race on asthma-related readmissions, with disease management and indoor environment partly mediating this effect (4.8% and 9.0% of the total effect, respectively).^128^

Socioeconomic inequalities in asthma outcomes are largely cited to be driven by inequalities in access to and quality of asthma care (for example, inadequate asthma reviews and inhaler technique checks, lower referral rates to specialist care), compromised asthma management (for example, lower ratio of controller to total prescribed asthma medication, excessive use of reliever medication, lower health literacy), exposure to environmental tobacco smoke, poor quality housing and inequalities in the physical environment (for example, increased exposure to air pollution and lack of access to green space), poor diet, and obesity.^70^ Additionally, known inequalities in uptake of influenza and other vaccines^129^, ^130^, ^131^ will influence the frequency and severity of respiratory infections, which are common triggers for asthma exacerbations.^117^^,^^132^ However, there is a clear need for studies examining the actual role and relative importance of these potential drivers, including how these vary across the life course and geographically, to ensure more effective targeting of interventions. While unhealthy diet and lifestyle patterns show fairly consistent social patterning across European populations, albeit to varying degrees, the social patterning of environmental exposures such as air pollution and access to green space appears more context-dependent.^109^

### How does asthma's heterogeneity affect pathways to social inequalities?

Given that different asthma subtypes appear to differ in their social patterning as well as their associated risk factors and pathophysiological mechanisms,^3^^,^^31^ there is also a need to consider how the drivers of inequalities differ for different subtypes, which to date has been little explored. This would similarly allow more effectively targeted interventions aimed at preventing the development and progression of specific asthma subtypes in underserved groups, as well as improving overall outcomes. With respect to the latter, it may be beneficial to consider different trajectories of exacerbations. For example, a study conducted in the Manchester Asthma and Allergy Study (MAAS) birth cohort used data-driven methods to map longitudinal trajectories of severe exacerbations from healthcare records, uncovering 2 trajectories of exacerbations: infrequent and early-onset frequent exacerbations.^59^ Each of these trajectories was associated with distinct early-life factors and asthma-related outcomes in adolescence, suggesting different underlying pathophysiological mechanisms. In particular, children in the early-onset frequent exacerbations trajectory had greater airway inflammation and significantly impaired lung function in adolescence, placing them at greater risk of respiratory conditions such as chronic obstructive pulmonary disease, as well as poorer cardiovascular, metabolic and mental health outcomes and premature death.^133^^,^^134^

## Summary

Childhood asthma disproportionately affects children from disadvantaged SECs, who are more likely to both develop persistent asthma and experience worse asthma outcomes. However, the extent of this social patterning appears to vary geographically and for different types of asthma.

Since children growing up in socioeconomically disadvantaged circumstances are at increased risk of several detrimental exposures over their life course that are also known risk factors for asthma, there are numerous potential pathways through which SECs may influence asthma and asthma outcomes, many of which are likely to be interdependent. While there has been some headway into understanding these complex pathways, further research is needed to fully understanding them. For example, the relative importance of the mediating pathways linking disadvantaged SECs and asthma diagnosis and asthma-related outcomes are likely to vary over the life course, with critical and sensitive periods during early life, but potentially also at other time points such as adolescence, which has not been explored. There is also a dearth of studies in LMICs examining longitudinal patterns of asthma symptoms and how these relate to SECs and related (potentially mediating) exposures. Since asthma subtypes are known to differ in their associated risk factors, the relative importance of the mediating pathways is also likely to vary across asthma subtypes, but this has also been little explored. A “one-size fits all approach” to tackling social inequalities in asthma will undoubtedly limit progress in understanding the complex mediating pathways driving disparities and identifying the most effective policy entry points, ultimately limiting our ability to address them effectively.

## Abbreviations

SECs, Socioeconomic circumstances; HICs, High-income countries; LMICs, Low- and middle-income countries; COPD, Chronic obstructive pulmonary disease; IgE, Immunoglobulin E; OR, Odds ratio; CI, Confidence interval; SABA, Short-acting beta-agonists; ICS, Inhaled corticosteroids; WHO, World Health Organisation; PM2.5, Particulate matter with a diameter less than 2.5 μm; MAAS, Manchester Asthma and Allergy Study.

## Ethics statement

Not applicable.

## Funding

In part supported through the NIHR Imperial Biomedical Research Centre. The views expressed are those of the author(s) and not necessarily those of the NIHR or the Department of Health and Social Care.

## Declaration of competing interest

Professor A Custovic reports personal fees from Novartis, personal fees from Sanofi, personal fees from Stallergenes Greer, personal fees from AstraZeneca, personal fees from Reacta Healthcare, personal fees from La Roche-Posay, outside the submitted work. Other authors have nothing to disclose.
